# The association between clinical prognostic factors and epidermal growth factor receptor-tyrosine kinase inhibitor (EGFR-TKI) efficacy in advanced non-small-cell lung cancer patients: a retrospective assessment of 94 cases with *EGFR* mutations

**DOI:** 10.18632/oncotarget.13787

**Published:** 2016-12-03

**Authors:** Jing-Hui Lin, Dong Lin, Ling Xu, Qiang Wang, Hui-Hua Hu, Hai-Peng Xu, Zhi-Yong He

**Affiliations:** ^1^ Department of Thoracic Medical Oncology, Fujian Provincial Cancer Hospital & Cancer Hospital Affiliated to Fujian Medical University, Fuzhou 350014, Fujian Province, China; ^2^ Group of Lung Cancer Treatment, Fujian Provincial Key Laboratory of Translational Cancer Medicine, Fuzhou 350014, Fujian Province, China

**Keywords:** non-small-cell lung cancer, epidermal growth factor receptor, epidermal growth factor receptor-tyrosine kinase inhibitor (EGFR-TKI), prognostic factor, retrospective analysis

## Abstract

**Objective:**

This study aimed to examine the association of clinical prognostic factors with epidermal growth factor receptor-tyrosine kinase inhibitor (EGFR-TKI) efficacy in advanced non-small-cell lung cancer (NSCLC) patients.

**Methods:**

The demographic and clinical characteristics of 94 patients with stage IV NSCLC were retrospectively reviewed, and the association between clinical factors and EGFR-TKIs efficacy was evaluated.

**Results:**

Of the 94 stage IV NSCLC patients enrolled in this study, a 74.5% objective response rate (ORR) and 97.9% disease control rate (DCR) were observed for EGFR-TKIs treatment, and a higher ORR was seen in patients with 0 and 1 ECOG scores than those with 2 or greater scores (*P* = 0.049). The subjects had a median PFS of 11 months and a median OS of 31 months after EGFR-TKIs treatment. ECOG score and timing of targeted therapy were factors affecting PFS, and ECOG score, smoking status and brain metastasis were factors affecting OS. In addition, ECOG score was an independent prognostic factor for PFS in stage IV NSCLC patients, and the patients with *EGFR* 19del mutation had a longer PFS than those with exon 21 L855R mutation (*P* = 0.003), while ECOG score and brain metastasis were independent prognostic factors for OS.

**Conclusions:**

The results of this study demonstrate that EGFR-TKI therapy results in survival benefits for *EGFR*-mutant advanced NSCLC patients, regardless of gender, smoking history, pathologic type, type of *EGFR* mutations, brain metastasis and timing of targeted therapy. ECOG score is an independent prognostic factor for PFS, and ECOG score and brain metastasis are independent prognostic factors for OS in advanced NSCLC patients.

## INTRODUCTION

Lung cancer is a malignant lung tumor with the highest incidence and mortality among all cancers worldwide [1], with a 5-year survival rate of only 16.8% [2]. Currently, epidermal growth factor receptor (EGFR) mutation is the most common type of gene mutations detected in Asian populations with lung cancer [3, 4], and EGFR is identified as the therapeutic target of EGFR tyrosine kinase inhibitors (TKIs) [5]. First-generation EGFR-TKIs have become the standard treatment for *EGFR*-mutant advanced non-small-cell lung cancer (NSCLC) [6, 7]. However, the demographic and clinical characteristics and the timing of administration of EGFR-TKIs, as well as brain metastasis, have been shown to affect the efficacy of the agents [8–10]. This retrospective study aimed to review the medical records of *EGFR*-mutant advanced NSCLC patients undergoing EGFR-TKIs treatment, so as to examine the association of clinical factors with EGFR-TKI efficacy in *EGFR*-mutant advanced NSCLC patients.

## RESULTS

### ORR in advanced NSCLC patients with EGFR-TKIs treatment

Among the 94 study subjects, there were 70 cases achieving PR and 22 cases achieving SD, with a 74.5% ORR and 97.9% DCR for EGFR-TKI therapy. The patients with 0 and 1 ECOG scores had a higher ORR than those with 2 or greater score (94.4% vs. 69.7%, *P* = 0.049), while there was no heterogeneity across the gender, age, smoking status, pathologic type, brain metastasis, timing of targeted therapy, or type of *EGFR* mutations (all *P* values > 0.05) (Table 1).

**Table 1:** Univariate analysis of demographic and clinical characteristics affecting the median PFS in stage IV NSCLC patients with EGFR-TKI therapy

Characteristics		No. of cases	Median PFS (months, 95% *CI*)	*P*
Overall		94	11 (10.1–11.9)	-
Gender	Male	49	12 (10.39–13.61)	0.304
	Female	45	11 (9.26–12.74)	
Age (years)	≤ 60	58	12 (11.07–12.93)	0.715
	> 60	36	10 (8.92–11.09)	
Smoking status	No	73	11 (10.03–11.97)	0.427
	Yes	21	10 (9.18–10.82)	
Pathologic type	Adenocarcinoma	86	11 (10.03–11.97)	0.713
	Non-adenocarcinoma	8	12 (9.23–11.77)	
Brain metastasis	Yes	33	11 (9.92–12.08)	0.963
	No	61	11 (9.66–12.34)	
Timing of targeted therapy	First line	28	15 (7.94–22.06)	0.04
	Second or higher line	66	11 (10.14–11.86)	
ECOG score	0–1	54	12 (10.29–13.71)	0
	≥ 2	40	8 (6.5–9.5)	
Type of *EGFR* mutation (64 cases)	*19del*	37	13 (10.05–15.95)	0.003
	*21L858R*	27	9 (7.53–10.47)	

### PFS in advanced NSCLC patients receiving EGFR-TKIs

**Figure 1:**
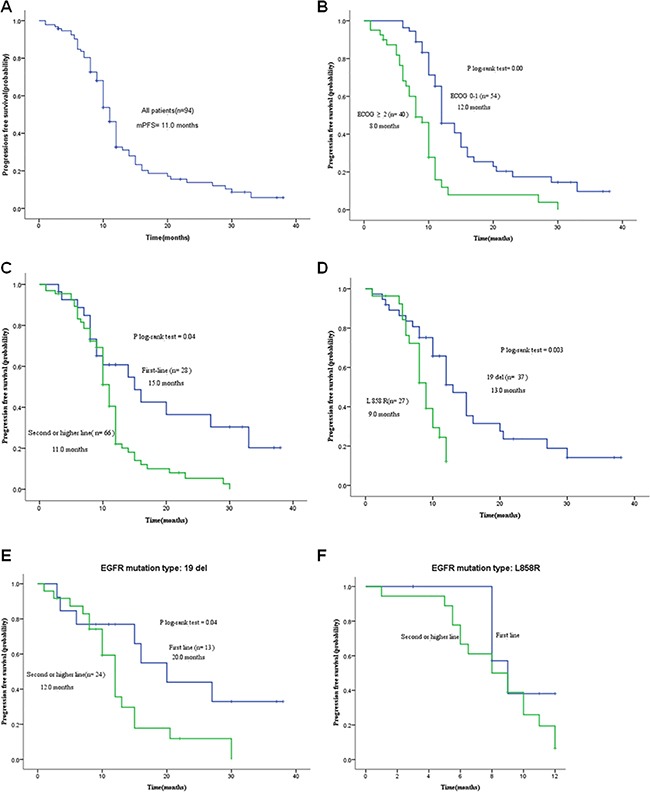
Kaplan-Meier curves of progression-free survival (PFS) **A**. The PFS of all study subjects (*n* = 94); **B**. PFS of patients with different ECOG scores; **C**. PFS of patients with various timing of EGFR-TKI treatment; **D**. PFS of patients with EGFR 19Del/L858R mutation; **E**. PFS of *EGFR*-mutant (deletions in exon 19) patients receiving various timing of EGFR-TKI treatment; **F**. PFS of *EGFR*-mutant (L858R) patients receiving various timing of EGFR-TKI treatment.

Following oral administration of EGFR-TKIs, the 94 subjects had a median PFS of 11 months (95% *CI*: 10.1–11.9 months) (Figure 1A), and 8 patients remained in continuous remission. Univariate analysis showed that the patients with 0 or 1 ECOG score had a longer PFS than those with 2 or higher score, and the patients receiving first-line targeted therapy had a longer PFS than those with second- or higher-line targeted therapies (Figures 1B and 1C); however, gender, age, brain metastasis and pathologic type did not significantly affect PFS in stage IV NSCLC patients (Table 1). Multivariate Cox regression analysis revealed ECOG score as an independent prognostic factor for PFS in stage IV NSCLC patients (Table 2), and the patients with *EGFR* 19del mutation had a longer PFS than those with exon 21 L855R mutation (13 vs. 9 months, *P* = 0.003) among the 64 *EGFR*-mutant NSCLC patients (Figure 1D–1F).

**Table 2:** Multivariate Cox regression analysis of clinical characteristics affecting the median PFS in stage IV NSCLC patients with EGFR-TKIs therapy

Affecting factors	β	SE	Wald	Sig.	Exp (B)	95% *CI*	Sig.
Timing of targeted therapy	0.591	0.308	3.668	0.055	1.805	0.986–3.304	0.04
ECOG score	0.936	0.253	13.722	0	2.549	1.554–4.182	0

### OS in advanced NSCLC patients receiving EGFR-TKIs

**Figure 2:**
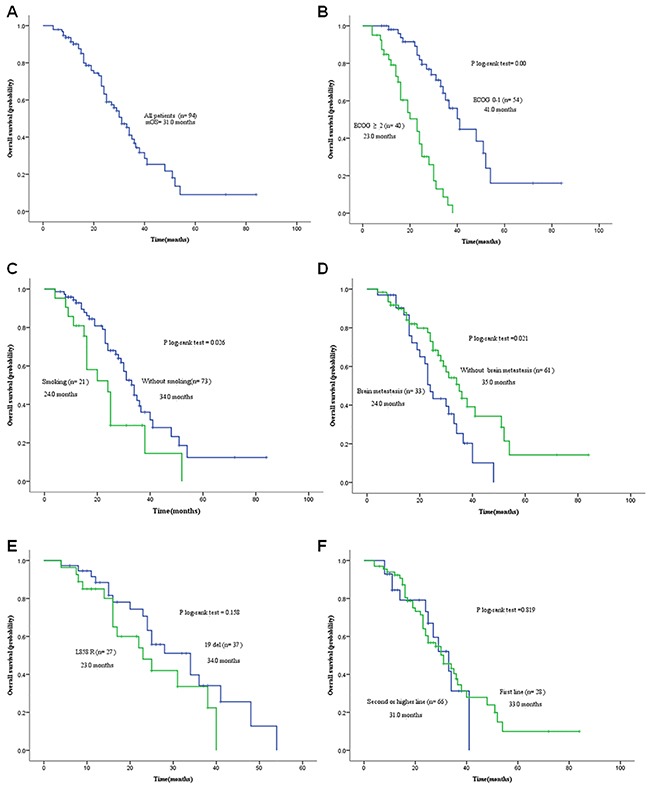
Kaplan-Meier curves of overall survival (OS) **A**. OS of all study subjects (*n* = 94); **B**. OS of patients with different ECOG scores; **C**. OS of patients with and without a history of smoking; **D**. OS of patients with and without brain metastasis; **E**. OS of patients with EGFR 19Del/L858R mutation; **F**. OS of patients receiving various timing of EGFR-TKI treatment.

The study subjects had a median OS of 31 months (95% *CI*: 26.18–35.82 months) after EGFR-TKIs treatment. There were 52 patients receiving follow up until the end of the study, and among the other 42 subjects that were still at follow-up, there were 14 cases with a survival of over 31 months, and 22 cases with a survival of over 24 months. The 1-, 2- and 3-year survival rates were 81.9%, 51.1% and 19.1% in the total study subjects, respectively (Figure 2A). Univariate analysis showed that the patients with 0 or 1 ECOG score had a longer OS than those with 2 or higher score (41 vs. 23 months, *P* = 0) (Figure 2B), and the patients with a history of smoking had a longer OS than those without a smoking history (34 vs. 24 months, *P* = 0.026) (Figure 2C), while the patients with brain metastases had a longer OS than those without brain metastases (35 vs. 24 months, *P* = 0.021) (Figure 2D). However, there was no heterogeneity across the gender, age, pathologic type, combination with chemotherapy or timing of targeted therapy (Table 3). Multivariate Cox regression analysis revealed ECOG score and brain metastasis as independent prognostic factors for OS in stage IV NSCLC patients (Table 4). In addition, no significant difference was found in the OS between the patients with *EGFR* 19del mutation and exon 21 L855R mutation (34 vs. 24 months, *P* = 0.158) (Figure 2E and 2F).

**Table 3:** Univariate analysis of demographic and clinical characteristics affecting the median OS in stage IV NSCLC patients with EGFR-TKIs therapy

Characteristics		No. of cases	Median OS (months, 95% *CI*)	*P*
Overall		94	31 (26.18–35.82)	-
Gender	Male	49	31 (17.46–44.54)	0.769
	Female	45	30 (24.26–35.75)	
Age (years)	≤ 60	58	31 (23.51–38.49)	0.66
	> 60	36	30 (20.92–39.08)	
Smoking status	No	73	34 (29.39–38.12)	0.026
	Yes	21	24 (16.86–31.14)	
Pathologic type	Adenocarcinoma	86	31 (25.89–36.11)	0.638
	Non-adenocarcinoma	8	24 (6.45–41.56)	
Brain metastasis	Yes	33	24 (20.93–27.07)	0.021
	No	61	35 (27.93–42.07)	
Timing of targeted therapy	First line	28	33 (24.02–41.98)	0.819
	Second or higher line	66	31 (22.06–39.94)	
ECOG score	0–1	54	41 (23.14–48.86)	0
	≥ 2	40	23 (15.82–30.18)	
Combination with chemotherapy	Yes	82	30 (23.6–36.41)	0.434
	No	12	34 (31.89–36.11)	
Type of *EGFR* mutation (64 cases)	*19del*	37	34 (22.63–45.37)	0.158
	*21L858R*	27	23 (13.09–32.9)	

**Table 4:** Multivariate Cox regression analysis of clinical characteristics affecting the median OS in stage IV NSCLC patients with EGFR-TKIs therapy

Affecting factors	β	SE	Wald	Sig.	Exp (B)	95% *CI*	Sig.
Smoking history	0.234	0.32	0.535	0.464	1.264	0.675–2.365	0.026
Brain metastasis	−0.838	0.297	7.949	0.005	0.432	0.241–0.774	0.021
ECOG score	1.81	0.342	28.092	0	6.112	3.129–11.938	0

## DISCUSSION

Previous randomized clinical trials have shown that EGFR-TKI is effective to increase the ORR, prolong the PFS and improve the quality of life relative to the standard chemotherapy in advanced *EGFR*-mutant NSCLC patients [11–14]. This retrospective study showed a 74.5% ORR, 97.9% DCR, a median PFS of 11 months and a median OS of 31 months in stage IV *EGFR* mutation-positive NSCLC patients treated of first- or higher-line EGFR-TKIs, which was similar to previous reports [15, 12–14]. In addition, the subjects with 0 and 1 ECOG score were found to have a higher ORR than those with 2 or greater scores, while other demographic and clinical characteristics showed no impact on ORR or DCR. Our findings demonstrate that gender, age, smoking status, pathologic type of NSCLC, type of *EGFR* mutation, timing of targeted therapy, and brain metastasis do not affect the short-term efficacy of EGFR-TKIs in stage IV NSCLC patients, and oral administration of EGFR-TKIs results in clinical benefits for advanced NSCLC patients harboring *EGFR* mutations.

As the most common types of *EGFR* gene mutation, exon 19 deletion mutation and exon 21 L858R mutation consist of 85% to 90% of all *EGFR* mutations [3]. The lung cancer patients harboring *EGFR* del19 mutation have been found to be more susceptible to EGFR-TKIs than those harboring exon 21 L855R mutation [16]. A meta analysis of 13 clinical trials showed that the stage IIIb/IV NSCLC patients with *EGFR* exon 19 deletion mutation had a longer PFS than those with L858R mutation at exon 21 following treatment with first-line EGFR-TKIs [17]. In the current study, no significant differences were observed in the ORR or DCR between stage IV NSCLC patients harboring *EGFR* 19del mutation and L858R mutation at exon 21, and univariate analysis showed a clear-cut effect of the *EGFR* mutation type on PFS (*P* = 0.003); however, the type of *EGFR* mutation was found to have no significant effect on OS (*P* = 0.158), which may be associated with the use of systemic chemotherapy in the study subjects. Exon 19 and 21 mutations may cause a difference in the sites of EGFR phosphorylation, resulting in the variation of its downstream signaling pathway. As compared to deletion mutation, high phosphorylation is detected on tyrosine residues encoded by the codon 845 in the L858 mutation at *EGFR* exon 21 [18], which may be responsible for the higher response to EGFR-TKIs in lung cancer patients harboring *EGFR* exon 19 mutation compared to those harboring exon 21 mutation.

The timing of EGFR-TKIs administration remains controversial in *EGFR*-mutant NSCLC patients [19–21]. A retrospective study showed that OS after erlotinib use was not different, whether used as first-, second- or third-line therapy in NSCLC patients [22]. Our findings showed that OS was not significantly different after EGFR-TKIs used as first-line or second/higher-line therapy (*P* = 0.819), indicating no effect of the timing of EGFR-TKIs on OS in advanced *EGFR*-mutant NSCLC patients. However, the PFS differed after EGFR-TKIs therapy given as first-line or second/higher-line therapy (*P* = 0.04), suggesting that the timing of EGFR-TKI use affected PFS in advanced *EGFR*-mutant NSCLC patients. Subgroup analysis revealed a longer PFS in patients given first-line EGFR-TKIs than in those receiving second- or higher-line EGFR-TKIs among the advanced NSCLC patients harboring *EGFR* exon 19 deletion mutation (*P* = 0.04), while PFS did not differed after EGFR-TKIs use, whether given as first-line, or second/higher-line therapy among the advanced NSCLC patients harboring L858R mutation at exon 21 (*P* = 0.229). To our knowledge, there was only one prospective randomized, controlled clinical trial to compare the efficacy of first-line erlotinib followed by second-line cisplatin/gemcitabine vs. first-line cisplatin/gemcitabine followed by second-line erlotinib for the treatment of advanced NSCLC patients to date [23]. In *EGFR* mutation-positive advanced NSCLC patients, first-line erlotinib followed by cisplatin-gemcitabine was found to show a better survival benefit over first-line chemotherapy followed by second-line erlotinib [24]. However, further randomized, controlled clinical trials are required to compare the clinical survival benefit from various combinations of EGFR-TKI and chemotherapy.

ECOG score has been found to remarkably affect the median survival in NSCLC patients, and it has been identified as a prognostic factor in NSCLC patients [25]. Previous studies have demonstrated that the advanced NSCLC patients with 0 or 1 ECOG score achieve better disease control and survival benefits from EGFR-TKI therapy than those with 2 or higher scores [25–27]. In the present study, we found a longer OS in stage IV NSCLC patients with an ECOG score of 0 and 1 than in those with an ECOG score of 2 or greater (*P* = 0), and ECOG score was identified as an independent prognostic factors for OS and PFS, indicating that ECOG score remains a prognostic factor in advanced *EGFR*-mutant NSCLC patients. It is therefore suggested that EGFR-TKIs should be given at early as possible in *EGFR* mutation-positive NSCLC patients, which may result in better survival benefits.

Brain metastasis has been identified as a poor prognostic factor for NSCLC, and the patients with brain metastasis was reported to have a median survival of 7 months and a 20% 1-year survival rate [28]. EGFR-TKI was found to partially penetrate the blood-brain barrier into the intracranial lesions in *EGFR* mutant NSCLC patients with brain metastasis [3, 29]. It has been reported that EGFR-TKI therapy results in a 56%–89% ORR at intracranial sites, a median PFS of 6.6 to 15.2 months and a median OS of 12.9 to 19.8 months in *EGFR* mutation-positive NSCLC patients with brain metastasis [30]. In this study, 35.1% of the study subjects were identified with brain metastasis at initial diagnosis, and 25 cases were given EGFR-TKI therapy combined with whole brain radiotherapy. The therapy achieved a 75.8% ORR and a median survival of 30 months, which was longer than previous reports [30]. Multivariate Cox regression analysis revealed brain metastasis as an independent prognostic factor for OS in stage IV NSCLC patients, and the patients with brain metastasis were found to have a shorter OS than those without brain metastasis (*P* = 0.021). Our findings demonstrate that the combination of EGFR-TKI and whole brain radiotherapy results in a longer OS than whole brain radiotherapy alone in NSCLC patients with brain metastasis, and brain metastasis is a prognostic factor for advanced NSCLC patients harboring *EGFR* mutation, which may be attributable to a higher risk of death. It has been reported that a higher concentration of EGFR-TKI is measured in the cerebrospinal fluid than in the plasma, thereby resulting in poor control of intracranial lesions [31]. Therefore, multiple treatments are encouraged to strengthen the management of metastatic brain lesions in *EGFR* mutation-positive NSCLC patients, such as increase of EGFR-TKI doses, administration of new-generation EGFR-TKIs to increase the drug concentration in the cerebrospinal fluid, and optimized combination of EGFR-TKI and whole brain radiotherapy, in order to extend OS in *EGFR* mutant NSCLC patients with brain metastasis.

Best supportive care (BSC) interventions, such as nutritional support, pain control, palliative brain radiotherapy, palliative bone radiotherapy and traditional Chinese medicine [32], have been proved to be beneficial for OS in cancer patients [33–35]. In this study, the NSCLC patients received various combinations of the BSC packages, which may result in survival benefits. The effect of BSC on EGFR-TKI efficacy cannot be completely excluded; however, we did not examine the effectiveness of BSC in the survival of advanced NSCLC patients. Further studies are required to compare the effectiveness of BSC alone, BSC plus EGFR-TKI, and EGFR-TKI alone in the survival of advanced NSCLC patients.

In conclusion, the results of the present study demonstrate that EGFR-TKI therapy results in survival benefits for *EGFR*-mutant advanced NSCLC patients, regardless of gender, smoking history, pathologic type, type of *EGFR* mutations, brain metastasis and timing of targeted therapy. ECOG score is an independent prognostic factor for PFS, and ECOG score and brain metastasis are independent prognostic factors for OS in advanced NSCLC patients. More randomized, controlled clinical trials are required to investigate the timing of EGFR-TKI treatment for advanced NSCLC patients.

## MATERIALS AND METHODS

### Subjects

A total of 94 stage IV NSCLC patients receiving oral administration of EGFR-TKIs (erlotinib, gefitinib or icotinib) in the Cancer Hospital Affiliated to Fujian Medical University during the period from February 2012 through February 2015 were enrolled in this study. Definite diagnosis was made by pathologic or cytological examinations, and all patients had measurable cancer lesions and complete clinical records. The study subjects consisted of (1) 65 *EGFR*-mutant NSCLC patients with oral administration of EGFR-TKIs; and (2) 29 NSCLC patients without *EGFR* mutations that were selected from the 53 patients showing a better response to EGFR-TKIs than the overall patient population (individuals with adenocarcinoma histology, females, and never-smokers) [36], including 25 patients with PR, and 4 patients with SD and a PFS of 10 months after EGFR-TKIs therapy. Mutation of the *EGFR* gene was detected using an amplification refractory mutation system (ARMS) [37]. The participants included 49 men and 45 women, and had a median age of 58 years (range, 33 to 82 years). There were 58 cases at ages of 60 years or less, and 36 cases aged over 60 years, and 73 cases without a smoking history and 21 cases with a history of smoking. Adenocarcinoma was detected in 86 cases and non-adenocarcinoma was found in 8 cases. In addition, there were 37 cases with *EGFR* *19del* mutation, 27 cases with exon *21 L858R* point mutation and one case with exon *18 G719X* mutation. According to the TNM staging system for NSCLC [38], all patients were identified as staged IV, and had metastases to multiple sites, including 33 cases with brain metastasis and 61 cases without brain metastasis. Of the total study subjects, 12 patients had never received chemotherapy, and 82 patients had received chemotherapy, including 7 cases undergoing first-line targeted therapy and 75 cases undergoing second- or higher-line therapy (Table 5). There were 77 cases given chemotherapy regimens containing a pemetrexed-platinum combination.

**Table 5:** Efficacy of EGFR-TKIs therapy in stage IV NSCLC patients with various demographic and clinical features

Characteristics		No. of cases	ORR (%)	*P*	DCR (%)	*P*
Overall		94	74.5	-	97.9	-
Gender	Male	49	77.4	0.875	100	0.884
	Female	45	77.8		95.6	
Age (years)	≤ 60	58	81	0.513	98.3	1
	> 60	36	63.9		97.2	
Smoking status	No	73	78.1	0.697	97.3	1
	Yes	21	61.9		100	
Pathologic type	Adenocarcinoma	86	76.7	0.559	97.7	1
	Non-adenocarcinoma	8	50		100	
Brain metastasis	Yes	33	75.8	1	97	1
	No	61	73.8		98.4	
Timing of targeted therapy	First line	28	85.7	0.612	100	1
	Second or higher line	66	69.7		96.7	
ECOG score	0–1	54	94.4	0.049	100	0.883
	≥ 2	40	47.5		95	
Type of *EGFR* mutation (64 cases)	*19del*	37	70.2	1	97.3	1
	*21L858R*	27	66.7		96.3	

ORR, objective response rate; DCR, disease control rate.

### Treatment regimen

All subjects were administered orally with erlotinib 150 mg QD (*n* = 82), gefitinib 250 mg QD (*n* = 8) or icotinib 125 mg TID (*n* = 4), until disease progression or intolerance to adverse events. The patients with PD were given first- or higher-line chemotherapy, and those that still had PD were orally administered with EGFR-TKIs. In addition, 25 out of the 33 patients with brain metastasis were given whole brain radiotherapy. During the treatment, no antacids were administered to ensure the normal absorption of EGFR-TKIs.

### Evaluating the response to EGFR-TKIs treatment

The response to EGFR-TKIs was evaluated one month post-treatment using the Response Evaluation Criteria in Solid Tumors (RECIST) version 1.1 [39], which was classified into complete response (CR), PR, SD and PD. CT scan was performed once every two months for evaluating the EGFR-TKIs efficacy in patients achieving stable or effective response, and the highest response was recorded. ORR was defined as the sum of CR rate (CRR) and PR rate (PRR), while disease control rate (DCR) was defined as the sum of CRR, PRR and rate of SD (SDR).

### Estimation of survival

PFS was defined as the duration from oral administration of EGFR-TKIs to disease progression or death, and OS was defined as the duration between the definite diagnosis and death or the end of the follow-up.

### Follow up

All 94 patients were followed up through the visits to the hospital for re-examinations or telephone until death or September 2015. The study subjects had a median follow-up period of 10 months (range, 7 to 43 months), with a 100% follow-up seen. During the follow-up period, 52 deaths occurred.

### Ethical statement

This study was approved by the Ethical Review Committee of the Cancer Hospital Affiliated to Fujian Medical University (approval no. FJZLYY2015-0219). Signed informed consent was obtained from all participants or their guardians following a detailed description of the purpose of the study.

### Statistics

All statistical analyses were performed using the statistical software SPSS version 22.0 (SPSS, Inc.; Chicago, IL, USA). The survival was estimated with the Kaplan-Meier method, and univariate analyses were performed with a log-rank test, while multivariate analyses were done using a Cox regression model. Differences of proportions were tested for statistical significance with chi-square test, with a *P* value < 0.05 considered statistically significant.
